# Cobalt diselenide nanobelts grafted on carbon fiber felt: an efficient and robust 3D cathode for hydrogen production†
†Electronic supplementary information (ESI) available: Experimental section, characterization, electrocatalytic study, and comparison of the literature catalytic parameters of various non-noble 3D HER electrocatalysts. See DOI: 10.1039/c5sc01335f



**DOI:** 10.1039/c5sc01335f

**Published:** 2015-05-18

**Authors:** Ya-Rong Zheng, Min-Rui Gao, Zi-You Yu, Qiang Gao, Huai-Ling Gao, Shu-Hong Yu

**Affiliations:** a Division of Nanomaterials and Chemistry , Hefei National Laboratory for Physical Sciences at Microscale , Collaborative Innovation Center of Suzhou Nano Science and Technology , Department of Chemistry , Institution University of Science and Technology of China , Hefei , Anhui 230026 , P. R. China . Email: shyu@ustc.edu.cn

## Abstract

Design and fabrication of low-cost, highly efficient and robust three-dimensional (3D) hierarchical structure materials for electrochemical reduction of water to make molecular hydrogen is of paramount importance for real water splitting applications. Herein, a 3D hydrogen evolution cathode constructed by *in situ* growing of cobalt diselenide nanobelts on the surface of commercial carbon fiber felt shows exceptionally high catalytic activity with 141 mV overpotential to afford a current density of 10 mA cm^–2^, and a high exchange current density of 5.9 × 10^–2^ mA cm^–2^. Remarkably, it also exhibits excellent catalytic stability, and could be used for more than 30 000 potential cycles with no decrease in the current density in 0.5 M H_2_SO_4_. This easily prepared 3D material with excellent electrocatalytic performance is promising as a realistic hydrogen evolution electrode.

## Abstract 2

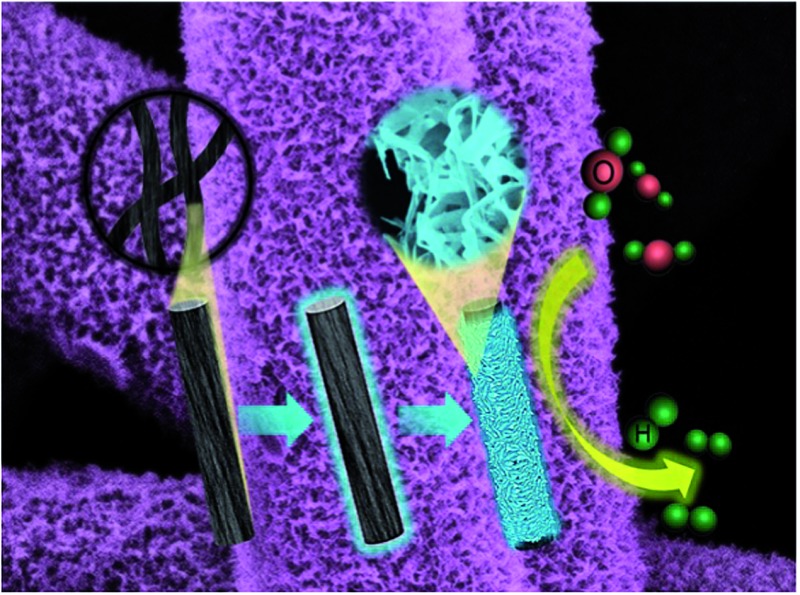
An easily scaled-up 3D CoSe_2_/CFF hierarchical electrode has been developed as a highly active and stable hydrogen evolution reaction cathode.

## Introduction

As a progressive energy carrier, H_2_ has attracted tremendous attention due to its potential as an alternative to fossil fuels.^1,2^ Currently, global hydrogen production mostly relies on natural gas, oil and coal, only a tiny part is from water electrolysis.^3^ Hence, energetically developing solar energy water splitting is critical for large-scale hydrogen technology. Hydrogen evolution reaction (HER) is a key process in photo/electrochemical water splitting, which needs an efficient and robust catalyst.^4^ Currently, platinum-based materials are the best HER catalysts, while the high-cost and scarcity of Pt heavily hinder its widespread use.^5^ Numerous efforts have been devoted to develop alternative HER electrocatalysts so far, including transition-metal chalcogenides (TMCs),^6–8^ phosphates,^9,10^ carbides,^11,12^ nitrides,^13–15^ and molecular catalysts,^16,17^ also some 3D HER electrodes such as molybdenum disulfide (MoS_2_)/fluorine-doped tin oxide,^18^ MoS_*x*_/graphene protected Ni foams,^19^ and CoSe_2_ nanoparticles (NPs) grown on carbon paper.^20^ Despite significant progress, preparing active, stable and cheap HER electrode materials remains a big challenge.

In the past years, we have explored new catalysts from TMCs and found that some TMCs possess decent HER and oxygen evolution reaction (OER) activities.^21–24^ Among which mesostructured CoSe_2_/DETA (DETA = diethylenetriamine) NBs and resultant hybrid materials exhibit high catalytic performances for both HER and OER. Very recently, we found that the HER activity and stability of CoSe_2_ NBs can be greatly enhanced after coating MoS_2_ on their surface due to the strong synergistic effect and increased catalytic sites.^25^ Therefore, as a multi-functional catalyst and an excellent substrate material, integrating the individual 2D CoSe_2_ NBs into a macroscopic 3D structure is of significant importance for practical application in energy conversion systems. Carbon fiber felt (CFF) has been used as the support material, due to the excellent mechanical strength, high conductivity, light-weight 3D structure, good corrosion resistance in harsh conditions, and large specific surface area as well as conductivity for intermediation transport.^26–30^

Herein, we report an economic, facile, and easily scaled-up method to prepare a CoSe_2_ NBs grafted CFF 3D architecture electrode (denoted as CoSe_2_/CFF). Remarkably, the 3D CoSe_2_/CFF electrode exhibits an extremely high stability under acidic conditions with a small onset potential of 96 mV *vs.* RHE, a Tafel slope of 68 mV per decade, and a large exchange current density of 5.9 × 10^–2^ mA cm^–2^, which outperforms those of a nanostructured MoS_2_ catalyst,^8^ MoS_2_/graphene hybrid,^31^ and other common non-precious metal catalysts.^32–34^ All these results strongly demonstrate the promise of a cheap, efficient and robust HER cathode based on CoSe_2_/CFF material.

## Results and discussion

**Fig. 1:**
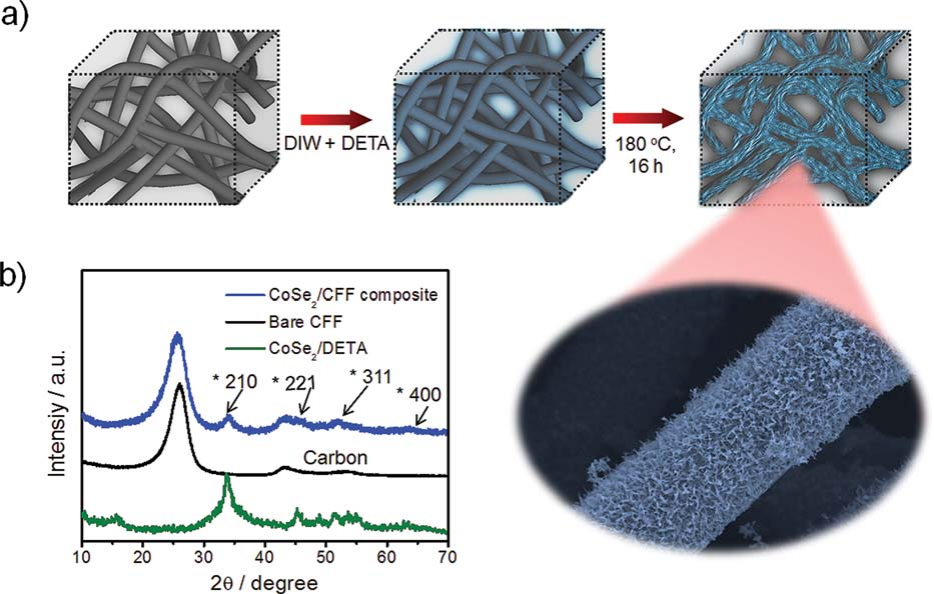
(a) Schematic illustration of the preparation of the CoSe_2_/CFF composite; (b) XRD patterns of CoSe_2_/DETA NBs, bare CFF and the CoSe_2_/CFF composite.

The porous commercial 3D CFF consists of ∼10 μm carbon fibers with a highly textured surface (ESI, Fig. S1†), and the carbon fibers (prepared by thermal treatment of polyacrylonitrile fibers) have a number of functional groups such as –COOH, –CO, and –CN (Fig. S2†), which would be beneficial for nucleation and growth of the CoSe_2_ NBs due to the strong chemical adhesion. The 3D CoSe_2_/CFF was synthesized by a one-step method in a closed solvothermal system (Fig. 1a). The phase of the prepared CoSe_2_/CFF composite is characterized by X-ray diffraction (XRD), as shown in Fig. 1b. It is observed that the diffraction peaks of the CoSe_2_/CFF composite are at 34.2°, 46.4°, 51.7°, and 63.4°, which were indexed to the (210), (221), (311), and (400) reflections of cubic phase CoSe_2_ (JCPDS card no. 09-0234), respectively. Other diffraction peaks of CoSe_2_ are hard to distinguish due to the relatively weak diffraction intensity and low amount compared to the CFF substrate. The peaks at 26.4° and 43.9° indicate graphitized carbon content of the CFF substrate.

**Fig. 2:**
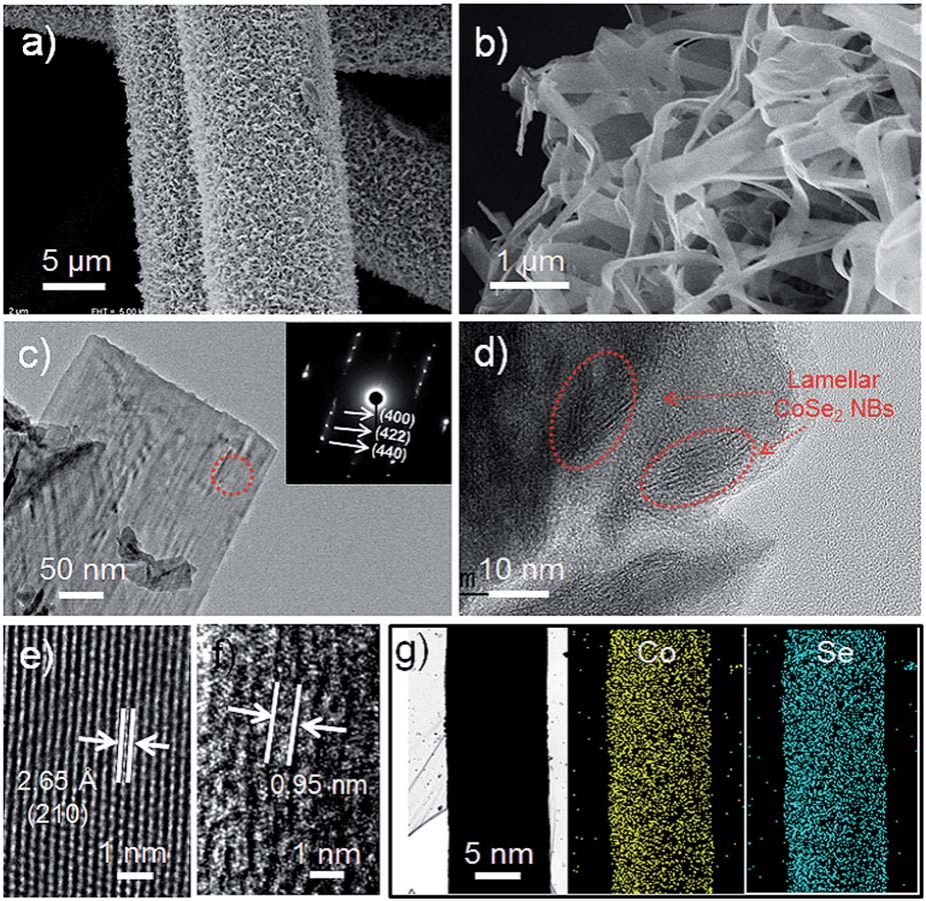
(a) and (b) SEM images with different magnifications of CoSe_2_/CFF composite; (c) and (d) TEM images with different magnifications of the CoSe_2_ NBs grafted on the surface of CFF. The inset in (c) shows the corresponding SAED pattern; (e) and (f) HRTEM images of the CoSe_2_/CFF composite; (g) STEM-EDS elemental maps of CoSe_2_/CFF composite overlap, Co (yellow) and Se (blue), respectively.

Fig. 2a shows a low magnification scanning electron microscopy (SEM) image of the CoSe_2_/CFF composite, which reveals that the entire surface of the CFF was uniformly covered by the densely packed CoSe_2_ NBs. By tuning the ratio of the raw materials and changing the volume of the reaction system, we can obtain different loading amounts from 4.37–22.43 wt% and various sizes (1–75 cm^2^) of CoSe_2_/CFF composites (Fig. S3 and S4†). Through the SEM image of the low loading (8.17 wt%) CoSe_2_/CFF composite, we find that the CoSe_2_ NBs with lengths of up to several micrometers and widths of *ca.* 200–800 nm are well grafted on the surface of the carbon fiber. It can be clearly seen that the grafted CoSe_2_ NBs still maintain their original flexible belt-like morphology (Fig. S5†). Transmission electron microscopy (TEM) images of the decorated CoSe_2_ NBs show the ultrathin lamellar nanostructure of the packed CoSe_2_ NBs (Fig. 2c and d), which would have advantages for the electro transform and the exchange of the intermediate during the catalytic reaction.^35^ The selected-area electron diffraction (SAED) pattern of the CoSe_2_ NBs (inset in Fig. 2c) shows the distinct diffraction spots index to the (400), (422) and (440) planes of the decorated CoSe_2_ NBs. The high resolution TEM (HRTEM) investigation in Fig. 2e shows the lattice fringe with a spacing of 2.65 Å can be assigned to the (210) plane of cubic phase CoSe_2_. The ordered lamellar CoSe_2_ NBs are also observed in Fig. 2d. We found that the interlayer distance is *ca.* 0.95 nm (Fig. 2f), which corresponds to the original CoSe_2_/DETA NBs.^36^ Scanning TEM energy dispersive X-ray spectroscopy (STEM-EDS) elemental mapping images of Co and Se for CoSe_2_ NBs are shown in Fig. 2g, further revealing that both Co and Se elements are uniformly distributed on the carbon fiber. Additionally, the CoSe_2_/CFF composite was also investigated using energy-dispersive X-ray spectroscopy (EDS) and X-ray photoelectron spectroscopy (XPS) (Fig. S6 and S7†). The interface between the CoSe_2_ NBs and CFF was detected in Fig. S8.† We found that fluffy nanosheets (NSs) were closely adhered on the CFF surface, and the dense CoSe_2_ NBs are grown upon these NSs. We investigated the growth process of the CoSe_2_ NBs on the CFF surface (Fig. S9†). In the beginning stage, the adsorbed Co and Se raw materials started to grow into NSs on the surface of the CFF. As the growth continued, more NSs nucleated on the surface, and the pre-obtained NSs would keep growing into belt structures, as the growth mechanism of the individual CoSe_2_/DETA NBs.^36^ Eventually, CoSe_2_ NBs covered the entire CFF surface, while also maintaining the CFF 3D architecture.

**Fig. 3:**
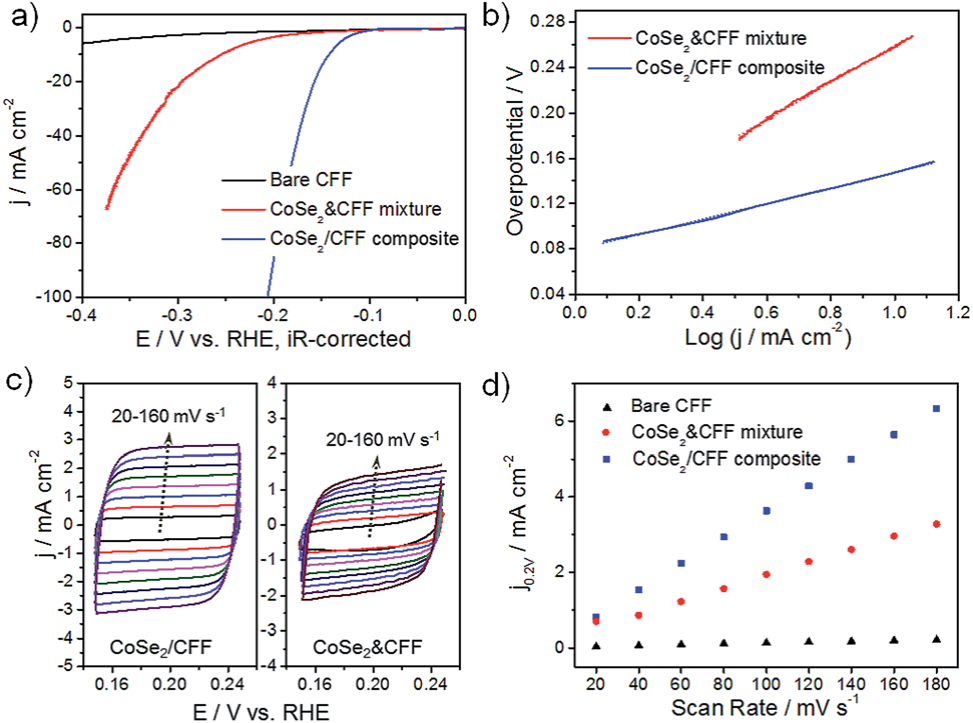
Electrochemical performance of CoSe_2_/CFF electrode in 0.5 M H_2_SO_4_. (a) iR-corrected polarization curves for HER on bare CFF, CoSe_2_ & CFF mixture and CoSe_2_/CFF composite, respectively; (b) Tafel plots and (c) cyclic voltammograms for CoSe_2_/CFF composite and CoSe_2_ & CFF mixture at different scan rates from 20 to 160 mV s^–1^, respectively; (d) the capacitive currents at 0.20 V *vs.* RHE as a function of scan rate for CoSe_2_ & CFF mixture and CoSe_2_/CFF composite.

The 3D CoSe_2_/CFF composite (loading amount: 8.17 wt%) was directly investigated as the working electrode in a typical three-electrode system for HER in 0.5 M H_2_SO_4_. Bare CFF and the same loading CoSe_2_ NBs and CFF physical mixture (denoted as CoSe_2_ & CFF) samples were also tested for comparison. As shown in Fig. 3a, the CoSe_2_ & CFF mixture shows a larger onset potential due to the aggregation of active materials and poor connection between CoSe_2_ and CFF, suggesting that the purely physical blend could not essentially enhance the catalytic activity (Fig. S10†). In contrast, the CoSe_2_/CFF composite exhibits a lower onset potential of 96 mV and greater cathodic current density. The overpotential of CoSe_2_/CFF required to drive the cathodic current density of 10 mA cm^–2^ is 141 mV, which is much lower than that of the CoSe_2_ & CFF mixture (252 mV). The bare CFF has poor HER activity in acidic solution. Additionally, the different loading catalysts suggest that the loading amount of 8.17 wt% is the optimal in this system (Fig. S11†).

The HER kinetics of the catalysts were probed by corresponding Tafel slopes (overpotential *versus* log current) (Fig. 3b). Under a specific set of conditions, the Tafel slopes of ∼120, ∼40, or ∼30 mV per decade will be achieved if the Volmer, Heyrovsky, or Tafel step is the rate-determining step, respectively. The experimentally measured Tafel slope of 68 mV per decade for the CoSe_2_/CFF composite indicates that the HER occurs by a Volmer–Heyrovsky mechanism.^23,37^ The strong chemical attachment and electrical coupling between the CFF and CoSe_2_ (Fig. S12†) enables an optimized electronic structure of CoSe_2_ upon its synergistic interaction with CFF that leads to faster HER kinetics. By contrast, a much higher Tafel slope of 123 mV per decade was observed for the CoSe_2_ & CFF mixture, which possibly stems from the fact that the active CoSe_2_ are just physically attached to the carbon fiber surface and thus no beneficial coupling effects were achieved. The HER catalyst inherent activity was further evaluated by the exchange current density (*j*_0_) based on the Tafel plot (Fig. S13†). As the key descriptor of the catalytic activity of an electrocatalyst, *j*_0_ could profoundly reflect the intrinsic electrochemical reaction rate.^38^ The *j*_0_ of 5.9 × 10^–2^ mA cm^–2^ for CoSe_2_/CFF outperforms most non-noble 3D HER electrocatalysts (see ESI Table S1†). Using the cyclic voltammetry (CV) method, we obtained the double layer capacitance (*C*_dl_), which is considered as an alternative approach to estimate the effective surface area (Fig. 3c and d).^33^ The *C*_dl_ of 17.2 mF cm^–2^ of the CoSe_2_/CFF electrode is much larger than that of CoSe_2_ & CFF (8.4 mF cm^–2^) and other reported 3D HER electrodes (Table S1†); pure CFF (0.56 mF cm^–2^) has negligible contribution to the capacitance. The reason is that for the CoSe_2_/CFF composite, active CoSe_2_ grafts uniformly onto every carbon fiber and forms a 3D architecture with advantageous holes, which allows better electrolyte and reactants/products transfer and thus a larger *C*_dl_ value. Since *C*_dl_ is proportional to the active surface area of the catalyst, the results suggest that the CoSe_2_/CFF is more effective in enlarging the catalytic surface area as compared to the CoSe_2_ & CFF mixture, and thus leading to the superior HER activity. The enhanced electrode kinetic factors (small onset potential of 96 mV and a Tafel slope of 68 mV per decade), large *j*_0_ of 5.9 × 10^–2^ mA cm^–2^ (∼1 order of magnitude lower than that of 0.71 mA cm^–2^ for Pt)^25^ and low impedance (Fig. S14†) indicate that the markedly faster HER kinetics compare favorably to the behavior of other non-noble HER electrocatalysts in acidic electrolyte, including the MoS_*x*_/graphene,^19^ CoSe_2_ NPs/carbon paper,^20^ and Co-doped FeS_2_/carbon nanotubes.^39^

**Fig. 4:**
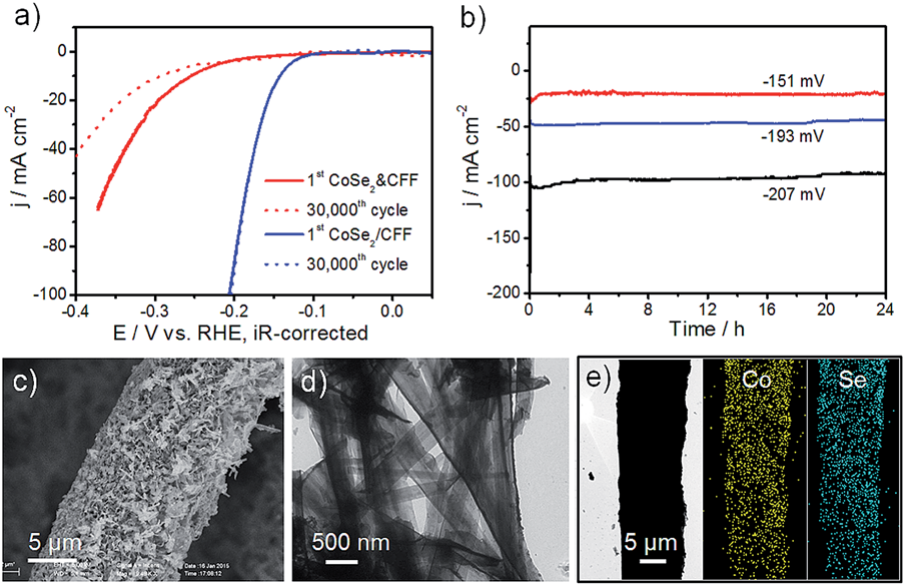
(a) ADT for CoSe_2_ & CFF and CoSe_2_/CFF electrodes before and after 30 000 potential cycles in 0.5 M H_2_SO_4_; (b) chronopotentiometry curves of CoSe_2_/CFF electrode during electrolysis over 24 h at fixed overpotential of –151, –193, and –207 mV (after iR correction), respectively; (c) and (d) SEM and TEM images of CoSe_2_/CFF after ADT; (e) STEM-EDS elemental maps of CoSe_2_/CFF after ADT, Co (yellow) and Se (blue), respectively.

High stability is a crucial factor for a good catalytic electrode. The accelerated durability tests (ADT) of CoSe_2_/CFF and CoSe_2_ & CFF electrodes were measured by taking continuous potential cycling at 100 mV s^–1^ for 30 000 cycles in 0.5 M H_2_SO_4_. As shown in Fig. 4a, the polarization curve of the CoSe_2_/CFF electrode after 30 000 cycles has negligible loss of the cathodic current, and the overlays almost coincide to the initial one. While the same testing leads to a large loss for the CoSe_2_ & CFF electrode. Compared with the chemically grafted CoSe_2_/CFF composite, CoSe_2_ NBs in the CoSe_2_ & CFF electrode are chaotically dispersed in the opening spaces of 3D CFF and physically attached to the CFF surface (Fig. S10†), which is more susceptible to suffering chemical corrosion during the ADT. Different loading CoSe_2_/CFF catalysts also have excellent stability in this system (Fig. S15†). In Fig. 4b, the time-dependent current density curves at fixed potentials also suggest the CoSe_2_/CFF electrode has superior durability over 24 h. Fig. 4c and d show the SEM and TEM images of the CoSe_2_/CFF after ADT; we found that the densely coated CoSe_2_ NBs still covered the CFF surface only with some aggregations. This suggests that the well-grafted CoSe_2_ on the CFF ensures the intimate contact and good chemical and mechanical adhesion, while the chemically resistant CFF can guarantee the advantageous 3D architecture. We therefore consider that the excellent stability of the CoSe_2_/CFF composite is contributed to by the strong chemical attachment of CoSe_2_ onto the porous 3D CFF that protects CoSe_2_ from growth, migration and aggregation during the continuous potential cycling process. STEM-EDS elemental map images indicate that Co and Se elements are still uniformly distributed on the CFF. XRD, EDS and XPS of the CoSe_2_/CFF after ADT were further employed to demonstrate the high durability of the CoSe_2_/CFF (Fig. S16†).

It is interesting to understand the intrinsic reasons for the enhanced activity and durability of the CoSe_2_/CFF. High-resolution Co 2p XPS spectra of individual CoSe_2_/DETA NBs and CoSe_2_/CFF show a dramatically increased electro-binding energy (∼0.9 eV) of Co 2p after growing CoSe_2_ NBs on the CFF surface (Fig. S12†), suggesting the presence of charge-transfer between CFF and CoSe_2_ NBs.^40^ The densely grafted CoSe_2_ NBs structure exposes more active sites, and the modified active site could sufficiently bond with adsorbed H* (a key intermediate during HER) for accelerating the proton–electron-transfer process.^38^ Furthermore, the highly opened 3D pore structure could provide more accessible active sites for water dissociation and facilitate the release of the generated gaseous H_2_ from the electrode surface. Meanwhile, it could offer a robust connection within the entire framework, enabling easy contact with the electrolyte.^41^ The excellent corrosion resistance ability of carbon fiber and strong chemical bonding with CoSe_2_ ensure the stable activity during the potential cycling process due to the 3D wrapping effect of the carbon fiber.^42^ Considering the comprehensive factors of the cost-effectiveness, high activity and corrosion resistance, the 3D CoSe_2_/CFF holds a promising prospect as a new HER cathode for large-scale hydrogen production.

## Conclusions

In summary, we report a facile method to prepare a highly active, stable 3D CoSe_2_/CFF hierarchical electrode, which is economic, facile and easily scaled-up. The prepared 3D CoSe_2_/CFF electrode is extremely robust and can be directly used as the HER cathode, which shows high activity for H_2_ evolution in acidic solution with an overpotential of 96 mV, Tafel slope of 68 mV per decade and a high exchange current density of 5.9 × 10^–2^ mA cm^–2^. The synergistic effect between CoSe_2_ and carbon fiber may contribute to the enhanced activity, and the 3D architecture framework leads to the high catalytic stability in the long-term operation. The easily prepared 3D CoSe_2_/CFF electrode holds a promise to replace the noble metal catalysts for electrochemical H_2_ production in viable water electrolytic systems.
