# Supplementary material for: Cobalt diselenide nanobelts grafted on carbon fiber felt: an efficient and robust 3D cathode for hydrogen production
Source: Chem Sci. 2015 May 18;6(8):4594–8. doi: 10.1039/c5sc01335f (PMC5500851; doi:10.1039/c5sc01335f)
Supplement: Supplementary file 1 [file SC-006-C5SC01335F-s001.pdf]

Electronic Supplementary Material (ESI) for Chemical Science.
This journal is © The Royal Society of Chemistry 2015

Supplementary Information

Cobalt diselenide nanobelts grafted on carbon fiber felt: an efficient and robust 3D cathode for hydrogen production†

Ya-Rong Zheng ‡, Min-Rui Gao ‡, Zi-You Yu, Qiang Gao, Huai-Ling Gao, and Shu-Hong Yu*

Division of Nanomaterials and Chemistry, Hefei National Laboratory for Physical Sciences at Microscale, Collaborative Innovation Center of Suzhou Nano Science and Technology, Department of Chemistry , Institution University of Science and Technology of China. Hefei, Anhui, 230026, P. R. China

E-mail: shyu@ustc.edu.cn

*Corresponding authors: shyu@ustc.edu.cn

 This journal is © The Royal Society of Chemistry [year]

Materials: Carbon fiber felt (thickness: 3 mm) was purchased from Gansu Haoshi Carbon Fiber Co., Ltd., China. All other chemicals are of analytical grade and were used as received without further purification.

Synthesis of $CoSe_2$/CFF composite. The $CoSe_2$ NBs decorated CFF was synthesized by a direct one-step method in a DETA-DIW solvothermal system as our previous work. Typically, 1 mmol $Co(CH_3COO)_2\cdot H_2O$ and 1 mmol Na_2SeO_3 were dissolved into mixture (40 mL) with a volume ratio of V_{DETA}/V_{DIW} = 2:1 (DIW: deionized water). Then, pieces of CFF (1 × 1 cm^2) were immerged in the solution for a 1 h, before that the CFF were cleaned in DIW and absolute ethanol mixture with sonication for 20 min. They were transferred into a 50 mL of Teflon-lined stainless autoclave, which was sealed and heated at 180 °C for 16h. The as-prepared were collected, washed with DIW and absolute ethanol for several times, and then dried under a vacuum at 60 °C for 6 h. The loading amount of $CoSe_2$ NBs on the CFF of $CoSe_2$ NBs/CFF was measured by microbalance.

Characterization. The samples were characterized by different analytic techniques. Scanning electron microscope (SEM) was operated on a field emission scanning electron microanalyzer (Zeiss Supra 40) at the acceleration voltage of 5 kV. Transmission electron microscope (TEM) images were obtained on a Hitachi H7650 transmission electron microscope with CCD imaging system on the acceleration voltage of 120 kV. High resolution TEM (HRTEM), selected area electron diffraction (SAED), the Energy disperse X-ray spectrum (EDS), and elemental mapping, were carried out on a JEM-ARM 200F Atomic Resolution Analytical Microscope on an acceleration voltage of 200 kV. X-Ray powder diffraction patterns (XRD) of the products were carried out on a Japan Rigaku DMax-γA rotation anode X-ray diffractometer equipped with graphite monochromatism Cu-Kα radiation (λ = 1.54178 Å). The X-ray photoelectron spectra (XPS) were recorded on an ESCALAB-MK-II X-ray photo-electron spectrometer using Mg Kα radiation exciting source (1253.6 eV). Fourier transform infrared (FT-IR) spectrum was carried on a Bruker Vector-22 FT-IR spectrometer from 4000 to 500 cm^{-1} at room temperature.

Electrocatalytic Study. Electrochemical measurements were performed at room temperature using a rotating disk working electrode made of glassy carbon (PINE, 5 mm diameter, 0.196 cm^2) connected to a Multipotentiostat (IM6ex, ZAHNER elektrik, Germany). Pt wire and saturated calomel electrode (SCE) were used as counter and reference electrodes, respectively. All potentials were converted to reversible hydrogen electrode (RHE) through RHE calibration described below.

Before the electrochemical measurement, the electrolyte (0.5 M H_2SO_4) was degassed by bubbling pure H_2 for 30 min. The polarization curves were obtained by sweeping the potential from -0.7 to -0.2 V vs. SCE, with a scan rate of 5 mV s^{-1}. The data were recorded after applying a number of potential sweeps until which were stable. The electrochemical impedance spectroscopy measurement was performed at overpotential 120 mV over a frequency range from 100 kHz to 20 mHz at a amplitude of the sinusoidal voltage of 5 mV and room temperature. The accelerated stability tests were performed in H_2-saturated 0.5 M H_2SO_4 at room temperature by potential cycling between -0.6 and -0.2 V vs. SCE at a sweep rate of 100 mV s^{-1} for 30,000 cycles. At the end of the cycles, the resulting electrode was used for HER polarization and CV curves at a sweep rate of 5 mV s^{-1}. All the polarization curves were corrected with iR-compensation.

This journal is © The Royal Society of Chemistry [year]

The polarization curves were re-plotted as overpotential (η) versus log current (log j) to get Tafel plots for assessing of the HER activities of investigated catalysts. By fitting the linear portion of the Tafel plots to the Tafel equation (η = b log(j) + a), the Tafel slope (b) can be obtained.

RHE calibration: In the all electrochemical tests, SCE was used as reference electrode. It was calibrated with regard to RHE. The calibration was performed in 0.5 M H_2SO_4 solution under H_2-saturated condition with a Pt-foil as the working electrode. CV curve was obtained at the scan rate of 1 mV s^{-1}, and the average potential at which the current crossed zero was regard as the thermodynamic potential for the hydrogen electrode reaction. In 0.5 M H_2SO_4 solution, $E_{RHE} = E_{SCE} + 0.278$ V.

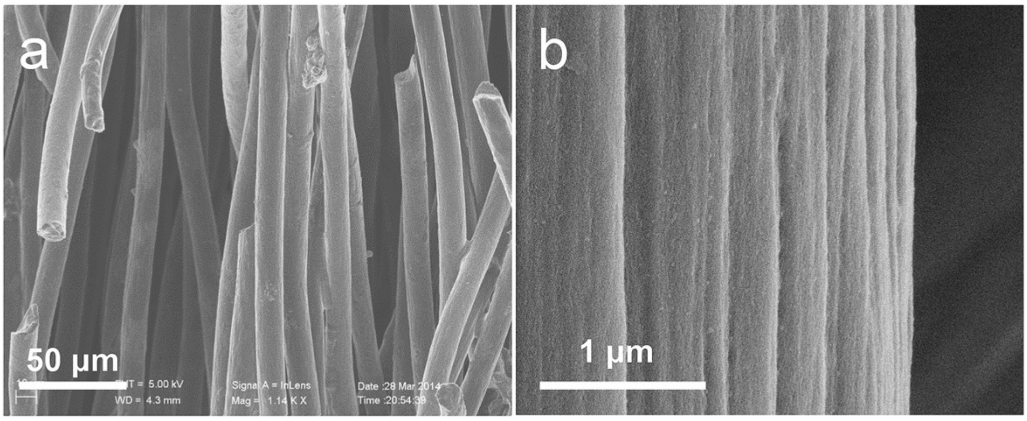

Fig. S1 SEM images of the bare CFF, which shows the texture surface of the carbon fiber with ~10 μm widths.

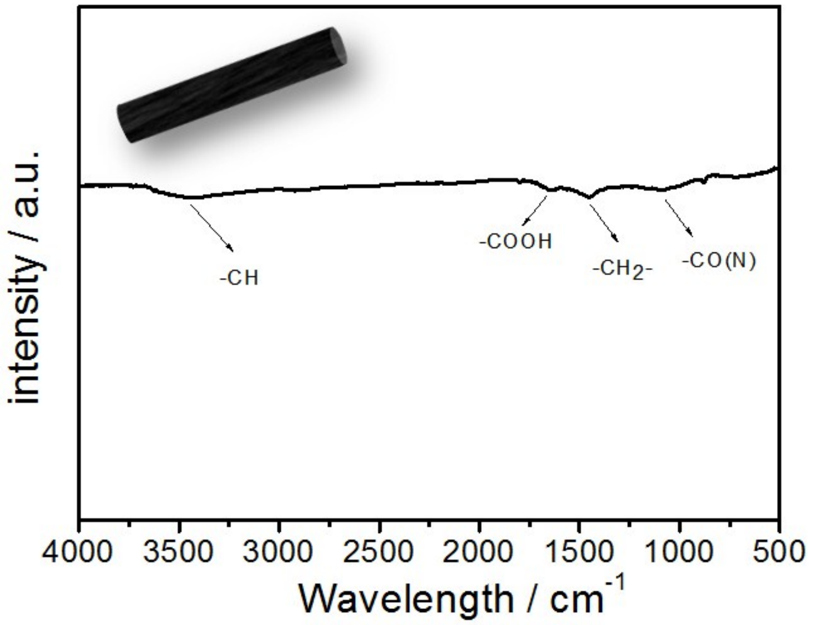

Fig. S2 Fourier transform infrared (FT-IR) spectrum of the bare carbon fiber felt. The copious –COOH, -CO, and -CN groups on the surface of CFF, which could serve as the nucleation sites to couple Co and Se precursors and grown into $CoSe_2$ NBs under suitable solvothermal reaction condition.

 This journal is © The Royal Society of Chemistry [year]

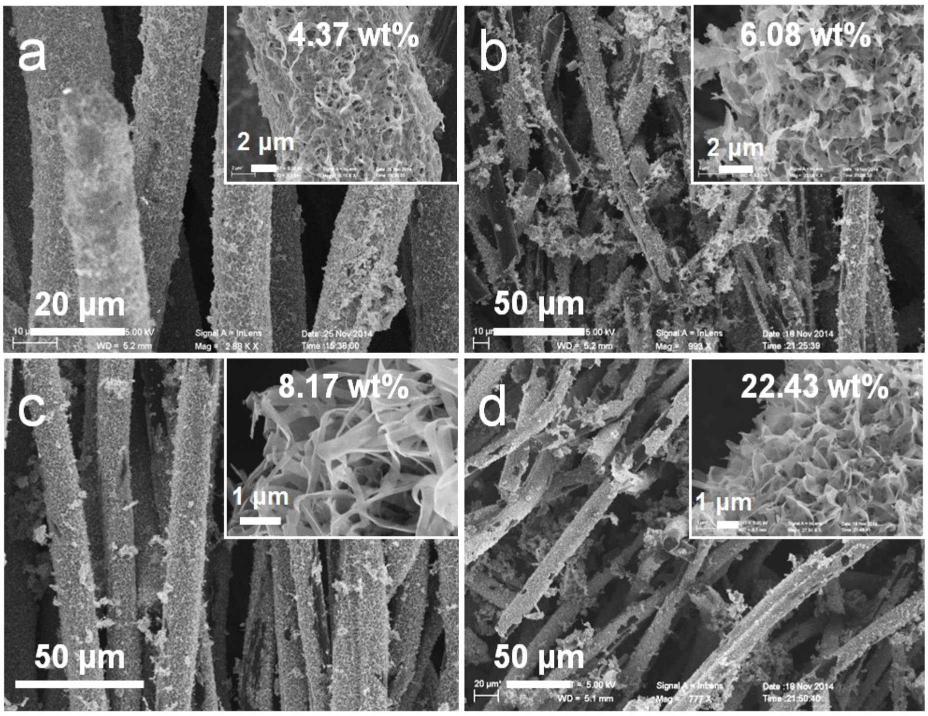

Fig. S3 SEM images of the different loading $CoSe_2$/CFF from 4.37 wt% to 22.43 wt%.

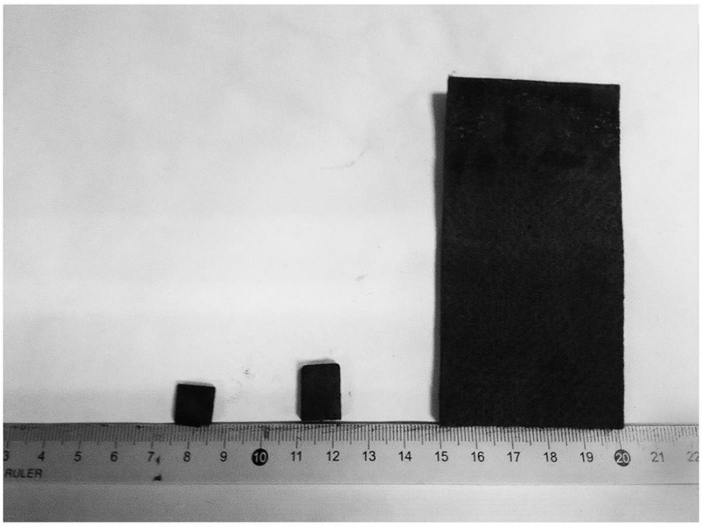

Fig. S4 Optical photograph of the various size $CoSe_2$/CFF composites from 1 cm^2 to 75 cm^2.

 This journal is © The Royal Society of Chemistry [year]

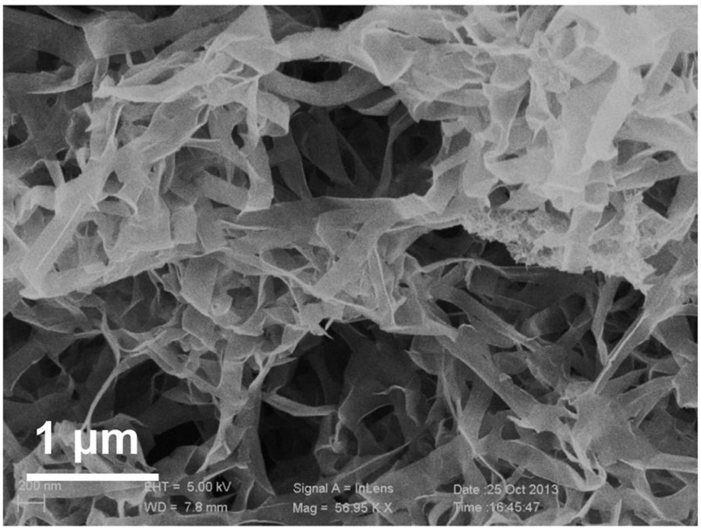

Fig. S5 SEM image of the freshly prepared $CoSe_2$/DETA NBs at 180 °C for 16h shows the $CoSe_2$ NBs with 100-800 nm and lengths up to several tens of micrometers with fexible, smooth and thin features.

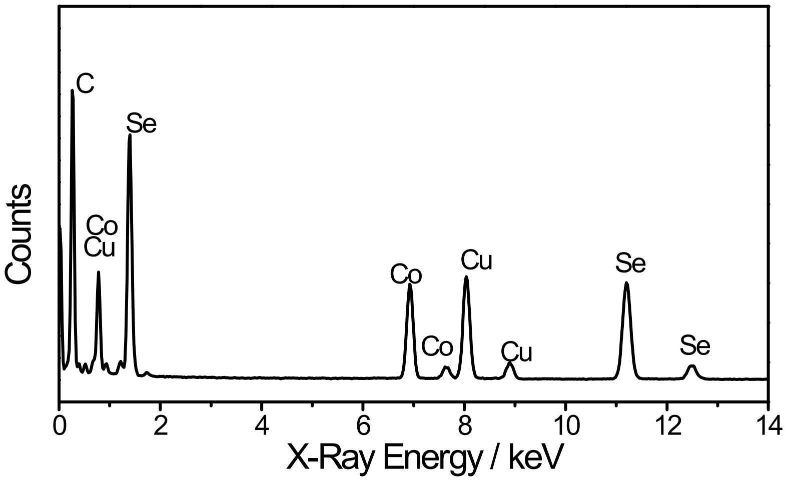

Fig. S6 EDX spectrum of the $CoSe_2$/CFF composite confirms the formation of $CoSe_2$/CFF composite, which was composed of the elements Co, Se and C, the presence of Cu peaks were emanated from the TEM grid.

 This journal is © The Royal Society of Chemistry [year]

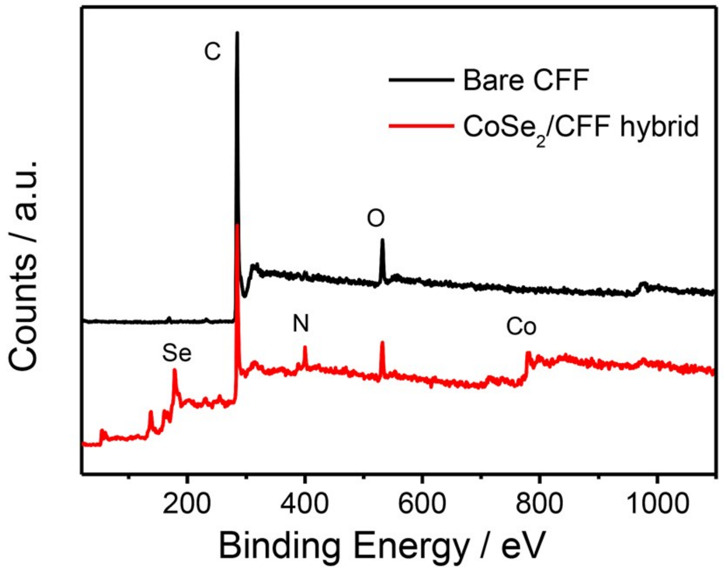

Fig. S7 XPS spectra for bare CFF and $CoSe_2$/CFF hybrid.

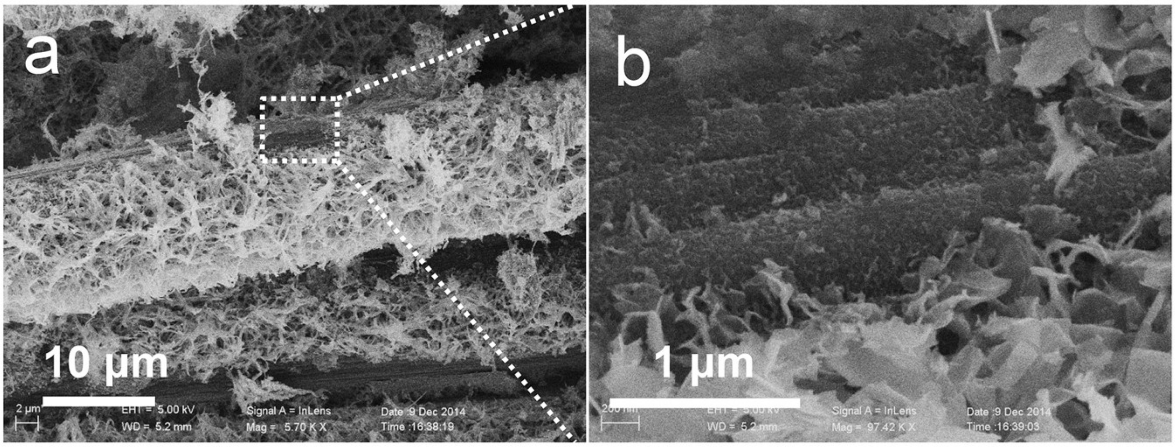

Fig. S8 SEM images of the interaction between $CoSe_2$ NBs and CFF.

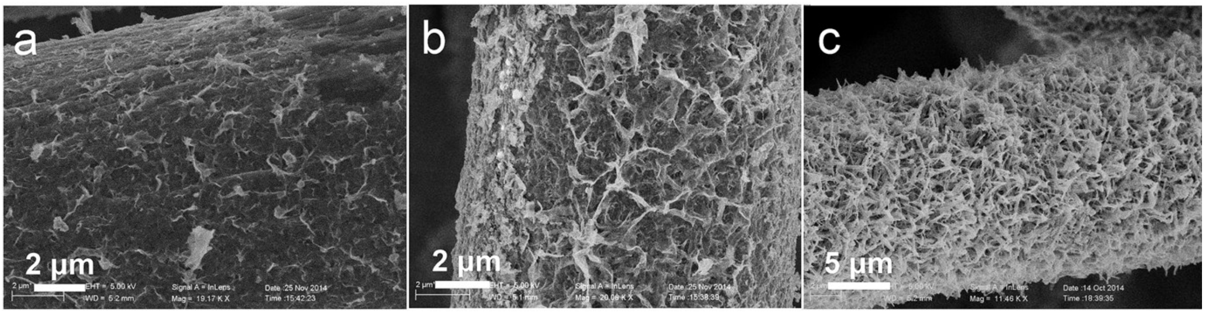

Fig. S9 SEM images of $CoSe_2$ /CFF at 180 ^{o}C for (a) 4h, (b) 12h, and (c) 16h.

This journal is © The Royal Society of Chemistry [year]

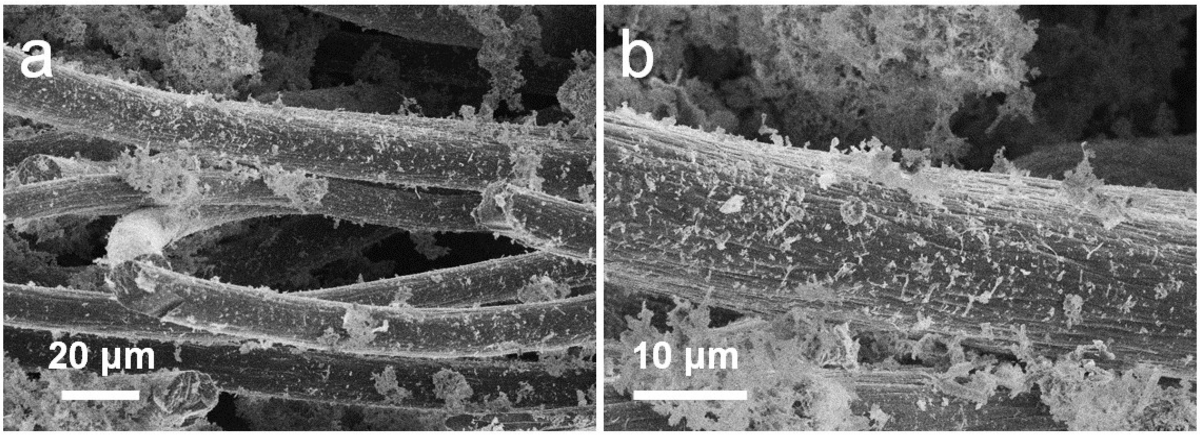

Fig. S10 SEM images of the $CoSe_2$ NBs and CFF physical mixture. A piece of 1 × 1 cm^2 CFF absorbs 8.17 wt% $CoSe_2$ (dispersed in 1 mL ethanol), then the mixture is dried under a vacuum at 60 °C.

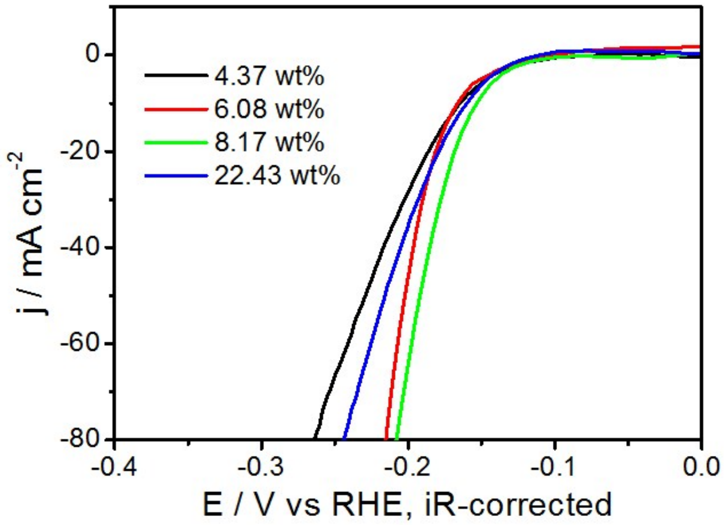

Fig. S11 IR-corrected polarization curves for HER on different loading $CoSe_2$/CFF electrodes, suggest that the loading amount of 8.17 wt% is the optimal in this system.

 This journal is © The Royal Society of Chemistry [year]

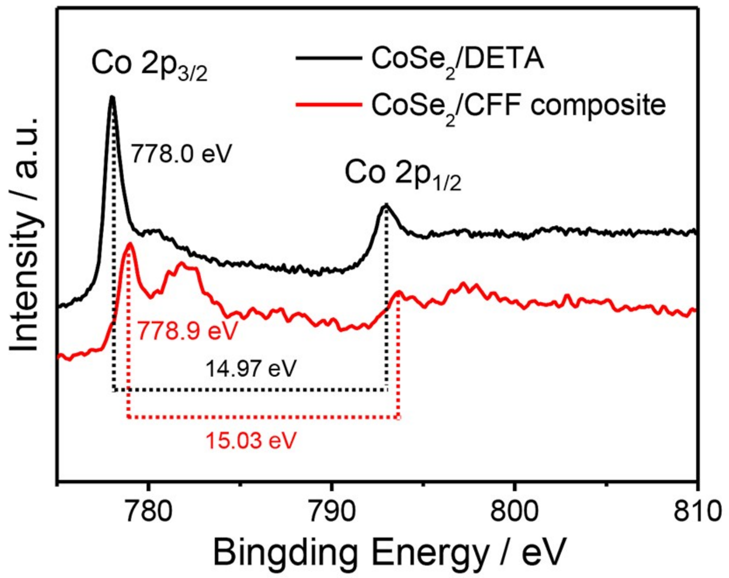

Fig. S12 High-resolution Co 2p XPS spectra for $CoSe_2$/DETA NBs and $CoSe_2$/CFF hybrid.

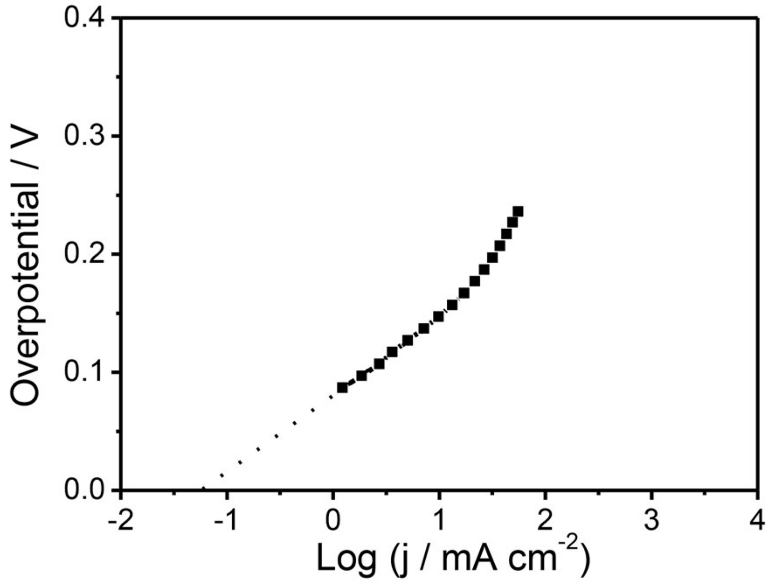

Fig. S13 Calculated exchange current density for $CoSe_2$/CFF in 0.5 M H_2SO_4.

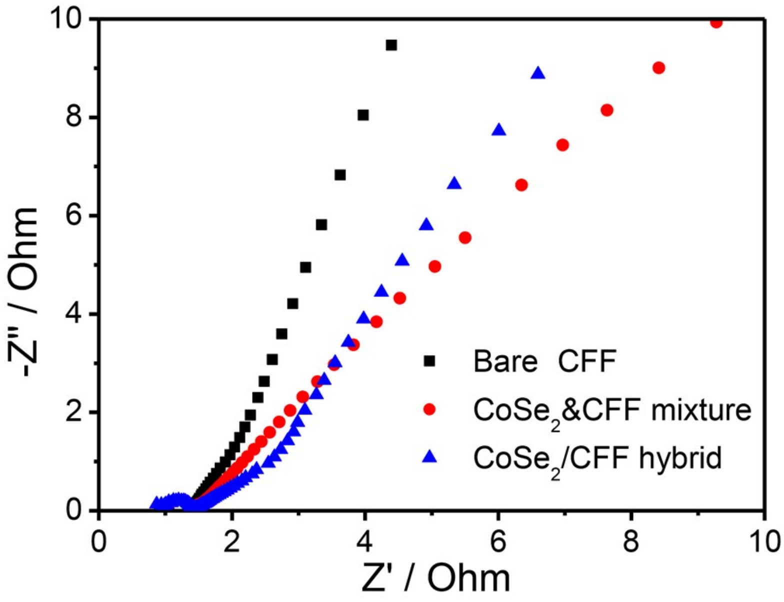

Fig. S14 Electrochemical impedance spectroscopy (EIS) Nyquist plots of bare CFF, $CoSe_2$&CFF mixture and $CoSe_2$/CFF hybrid. Z' is the real impendence and -Z'' is the imaginary impedance.

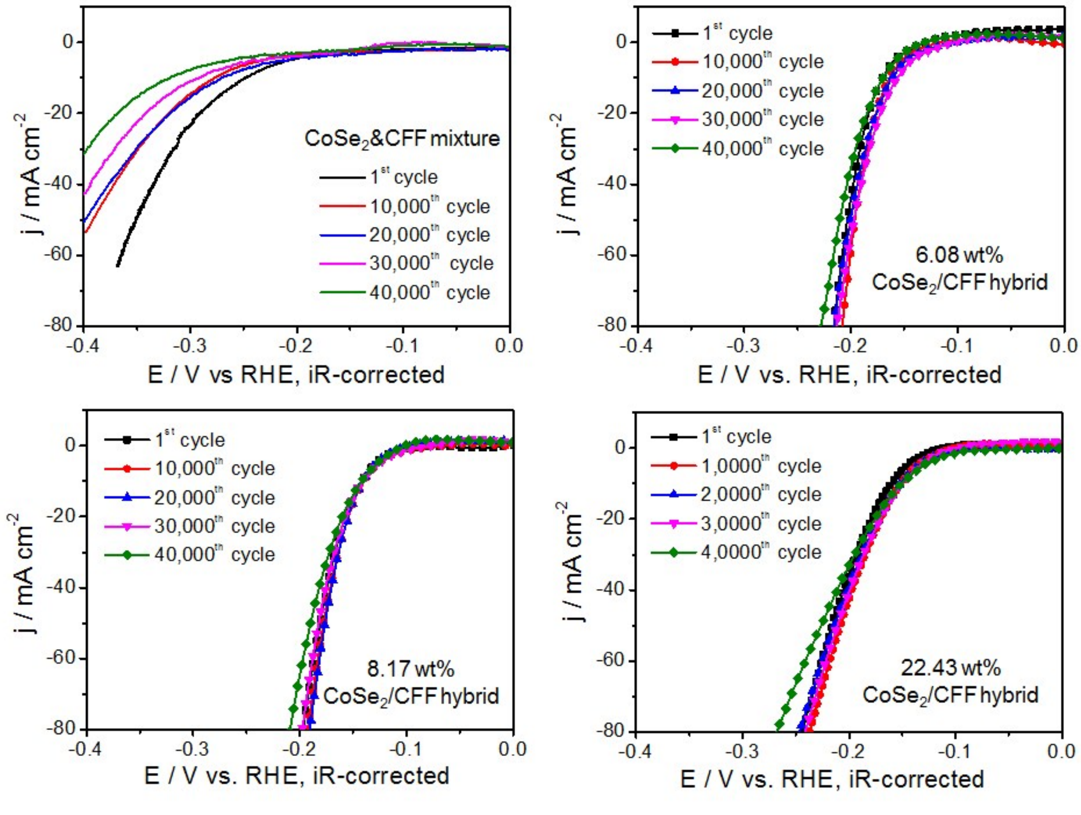

Fig. S15 ADT for $CoSe_2$&CFF mixture and different loading $CoSe_2$/CFF electrodes before and after each 10,000 potential cycles, which reveal that the $CoSe_2$ NBs decorated CFF process high durability in 30,000 potential cycles, and have little loss of current density after 40,000 cycles.

This journal is © The Royal Society of Chemistry [year]

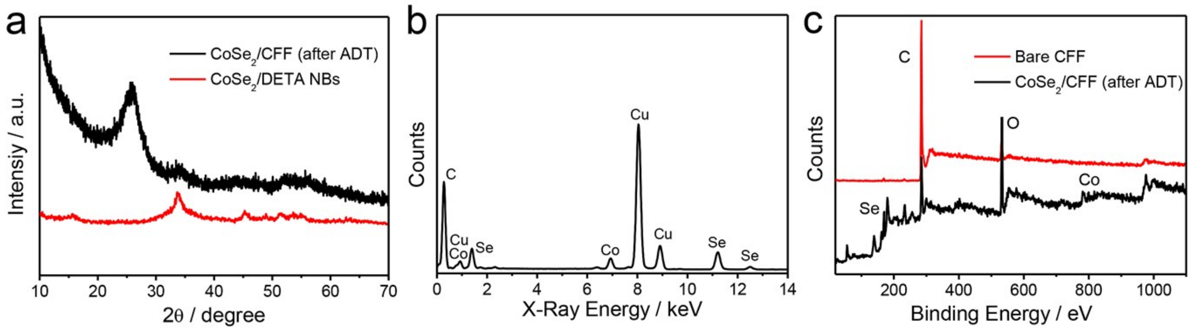

Fig. S16 (a) XRD, (b) EDS, and (c) XPS spectra of the $CoSe_2$/CFF after ADT.

 This journal is © The Royal Society of Chemistry [year]

Table S1. Comparison of catalytic parameters of various non-noble 3D HER electrocatalysts reported in literature (Electrolyte: 0.5 M H_2SO_4).

Catalyst/ Loading (mg cm^{-2})	Exchange current density (mA cm^{-2})	η @ j = 10 mA cm^{-2} (mV vs. RHE)	Tafel slope (mV dec^{-1})	ADT potential cycles	Double layer capacitance (mF cm^{-2})	Ref.
$CoSe_2$ NBs/CFF (4.3)	5.9×10^{-2}	141	68	30,000	17.2	This work
$CoSe_2$ NPs/CP (2.5-3.0)	4.9×10^{-3}	139	42	5,000	14.1	Ref. [1]
$MoSe_2$/CP (N/A)<br>WSe_2/CP (N/A)	3.8×10^{-4}<br>N/A	250<br>300	59.8<br>77.4	15,000<br>15,000	N/A<br>N/A	Ref. [2]
MoS_2/CC (N/A)	9.2×10^{-3}	159	50	2,000	N/A	Ref. [3]
CoP/CC (0.92)	0.288	67	51	1,000	N/A	Ref. [4]
CoS_2/CNT/ Graphene (1.15)	6.26×10^{-2}	142	51	500	N/A	Ref. [5]
Mo_xS/N-doped graphene (0.7)	N/A	140.6	105	1,000	N/A	Ref. [6]
MoS_2 film/Mo foil (8.5×10^{-3})<br>$MoSe_2$ film/Mo foil (13.5×10^{-3})	2.2×10^{-3}<br>2.0×10^{-3}	N/A<br>N/A	40<br>75	1,000<br>1,000	N/A<br>N/A	Ref. [7]
MoS_x/graphene/ Ni foam (5.01)	N/A	141	42.8	N/A	N/A	Ref. [8]
MoS_2/fluorine-doped tin oxide (0.06)	4.9×10^{-4}	195	50	N/A	131 (mC/cm^2)	Ref. [9]
CuP NWs/Cu foil (15.2)	0.18	161	67	3,000	77.8	Ref. [10]
FeP NA/Ti foil (3.2)	0.42	55	38	3,000	N/A	Ref. [11]
MoP-S/Ti foil (3.0)	0.57	64	50	1,000	14.3	Ref. [12]

 This journal is © The Royal Society of Chemistry [year]

Notes and references

1. D. S. Kong, H. T. Wang, Z. Y. Lu and Y. Cui, *J. Am. Chem. Soc.*, 2014, **136**, 4897-4900.
2. H. T. Wang, D. S. Kong, P. Johanes, J. J. Cha, G. Y. Zheng, K. Yan, N. A. Liu and Y. Cui, *Nano Lett.*, 2013, **13**, 3426-3433.
3. Y. X. Yan, B. Y.; Li, N.; Xu, Z.; Fisher, A.; Wang, X.; , *J. Mater. Chem. A*, 2015, **3**, 131.
4. J. Q. Tian, Q. Liu, A. M. Asiri and X. P. Sun, *J. Am. Chem. Soc.*, 2014, **136**, 7587-7590.
5. S. Peng, L. Li, X. Han, W. Sun, M. Srinivasan, S. G. Mhaisalkar, F. Cheng, Q. Yan, J. Chen and S. Ramakrishna, *Angew. Chem. Int. Ed.*, 2014, **53**, 12594-12599.
6. S. Chen, J. Duan, Y. Tang, B. Jin and S. Zhang Qiao, *Nano Energy*, 2015, **11**, 11-18.
7. D. S. Kong, H. T. Wang, J. J. Cha, M. Pasta, K. J. Koski, J. Yao and Y. Cui, *Nano Lett.*, 2013, **13**, 1341-1347.
8. Y. H. Chang, C. T. Lin, T. Y. Chen, C. L. Hsu, Y. H. Lee, W. Zhang, K. H. Wei and L. J. Li, *Adv. Mater.*, 2013, **25**, 756-760.
9. J. C. Kibsgaard, Z.; Reineche, B. N.; Jaramillo, T. F.;, *Nat. Mater.*, 2012, **11**, 963.
10. J. Tian, Q. Liu, N. Cheng, A. M. Asiri and X. Sun, *Angew. Chem. Int. Ed.*, 2014, **53**, 9577-9581.
11. P. Jiang, Q. Liu, Y. Liang, J. Tian, A. M. Asiri and X. Sun, *Angew. Chem. Int. Ed.*, 2014, **53**, 12855-12859.
12. J. Kibsgaard and T. F. Jaramillo, *Angew. Chem. Int. Ed.*, 2014, **53**, 14433-14437.

 This journal is © The Royal Society of Chemistry [year]
